# The Curious Case of the Nidovirus Exoribonuclease: Its Role in RNA Synthesis and Replication Fidelity

**DOI:** 10.3389/fmicb.2019.01813

**Published:** 2019-08-07

**Authors:** Natacha S. Ogando, Francois Ferron, Etienne Decroly, Bruno Canard, Clara C. Posthuma, Eric J. Snijder

**Affiliations:** ^1^Department of Medical Microbiology, Leiden University Medical Center, Leiden, Netherlands; ^2^Architecture et Fonction des Macromolécules Biologiques, Centre National de la Recherche Scientifique, Aix-Marseille Université, Marseille, France; ^3^European Virus Bioinformatics Center, Jena, Germany

**Keywords:** nidovirus, non-structural protein 14, exoribonuclease, N7-methyltransferase, proofreading, coronavirus

## Abstract

Among RNA viruses, the order *Nidovirales* stands out for including viruses with the largest RNA genomes currently known. Nidoviruses employ a complex RNA-synthesizing machinery comprising a variety of non-structural proteins (nsps). One of the postulated drivers of the expansion of nidovirus genomes is the presence of a proofreading 3′-to-5′ exoribonuclease (ExoN) belonging to the DEDDh family. ExoN may enhance the fidelity of RNA synthesis by correcting nucleotide incorporation errors made by the RNA-dependent RNA polymerase. Here, we review our current understanding of ExoN evolution, structure, and function. Most experimental data are derived from studies of the ExoN domain of coronaviruses (CoVs), which were triggered by the bioinformatics-based identification of ExoN in the genome of severe acute respiratory syndrome coronavirus (SARS-CoV) and its relatives in 2003. Although convincing data supporting the proofreading hypothesis have been obtained, from biochemical assays and studies with CoV mutants lacking ExoN functionality, the features of ExoN from most other nidovirus families remain to be characterized. Remarkably, viable ExoN knockout mutants were obtained only for two CoVs, mouse hepatitis virus (MHV) and SARS-CoV, whose RNA synthesis and replication kinetics were mildly affected by the lack of ExoN function. In several other CoV species, ExoN inactivation was not tolerated, and knockout mutants could not be rescued when launched using a reverse genetics system. This suggests that ExoN is also critical for primary viral RNA synthesis, a property that poorly matches the profile of an enzyme that would merely boost long-term replication fidelity. In CoVs, ExoN resides in a bifunctional replicase subunit (nsp14) whose C-terminal part has (N7-guanine)-methyltransferase activity. The crystal structure of SARS-CoV nsp14 has shed light on the interplay between these two domains, and on nsp14’s interactions with nsp10, a co-factor that strongly enhances ExoN activity *in vitro* assays. Further elucidation of the structure-function relationships of ExoN and its interactions with other (viral and/or host) members of the CoV replication machinery will be key to understanding the enzyme’s role in viral RNA synthesis and pathogenesis, and may contribute to the design of new approaches to combat emerging nidoviruses.

## Introduction

RNA viruses typically exhibit a high mutation frequency. This intrinsic biological property facilitates rapid adaptation of the virus to changing circumstances, a major contributor to the frequent outbreaks of mutated or newly emerging RNA viruses in humans, livestock, and other host organisms. The poor fidelity of RNA virus genome replication is attributed primarily to the fact that errors made by the viral RNA-dependent RNA polymerase (RdRp) go uncorrected. This lack of proofreading results in “quasispecies” populations of closely related viral genomes that are subject to continuous natural selection. As the accumulation of an excessive number of deleterious mutations can result in “error catastrophe,” the low fidelity of their replication is thought to have restricted genome size, which for most RNA virus families is (well) below 15 kilobases (Steinhauer et al., 1992; Drake and Holland, 1999; Eigen, 2002). This evolutionary trade-off between RNA virus genome size, replication fidelity, and adaptive capacity has been explored both from a fundamental perspective and in the context of antiviral drug development (Crotty et al., 2001). The balance between quasispecies diversity and viral fitness appears to be easily disturbed, suggesting that RNA viruses in general may operate close to their error threshold (Pfeiffer and Kirkegaard, 2005; Vignuzzi et al., 2006; Manrubia et al., 2010). Clearly, in the absence of countermeasures to reduce the overall error rate, similar issues would be expected upon the significant expansion of RNA virus genome size.

The largest RNA genomes currently known are all found in the order *Nidovirales*, an order of positive-stranded RNA (+ RNA) viruses that includes the coronavirus (CoV) family as its best-studied taxon. Recent nidovirus additions (Bukhari et al., 2018; Saberi et al., 2018) have increased the known upper limit of RNA genome size from just above 30 kb (for most CoVs) to more than 41 kb in a nidovirus identified in a planarian host, which was named planarian secretory cell nidovirus (PSCNV) (Saberi et al., 2018). About 15 years ago, during the in-depth bioinformatics analysis of the genome and proteome of the severe acute respiratory syndrome coronavirus (SARS-CoV), Alexander Gorbalenya and co-workers identified a 3′-5′ exoribonuclease (ExoN) signature sequence in a domain embedded in the replicase polyprotein of CoVs and other nidoviruses with a similarly large RNA genome, and speculated about its role as a proofreading enzyme in the evolution of such large nidovirus genomes (Snijder et al., 2003). Shortly after this ground-breaking discovery, ExoN activity was demonstrated biochemically for SARS-CoV (Minskaia et al., 2006) and – following its inactivation by reverse genetics – was indeed implicated in enhancing CoV replication fidelity (Eckerle et al., 2007). Subsequently, the enzyme was the subject of further virological, biochemical, structural, and genetics studies. Evidence strongly supporting the “proofreading exoribonuclease” hypothesis has now accumulated, in particular for SARS-CoV and murine hepatitis CoV (MHV) (Bouvet et al., 2012; Smith and Denison, 2012), and will be summarized below. At the same time, quite different observations were made for multiple other CoVs, highlighting the need for a more extensive experimental characterization of the importance and function of the unique ExoN domain, both within the CoV family and in other nidovirus subgroups.

ExoN acquisition by a nidoviral ancestor and the subsequent development of a beneficial interplay with the viral RNA RdRp (Subissi et al., 2014a; Ferron et al., 2018) are thought to have been key steps in relieving the constraints on genome size expansion in this virus lineage (Nga et al., 2011). Strikingly, the replication of arteriviruses, the nidovirus family with the smallest genome (12–16 kb), does not depend on the presence of an ExoN domain in the viral replicase (Snijder et al., 2003), suggesting they either diverged from other nidoviruses before ExoN acquisition or lost ExoN at a later stage of their evolutionary trajectory.

## The Amazing Diversity of Nidoviruses

The order *Nidovirales* currently comprises 88 formally recognized virus species of + RNA viruses, which are classified in nine virus families across seven different suborders (Siddell et al., 2019)^1^. These agents can infect a striking variety of vertebrate and invertebrate hosts, including mammals, birds, amphibians, fish, reptiles, arthropods, molluscs, and helminths. Additional nidovirus genome sequences continue to be described, due to the extensive metagenomics-based virus discovery efforts of the past decade (Saberi et al., 2018). Their adequate classification will undoubtedly require the creation of additional nidovirus taxa. Unfortunately, the biological and (possible) pathogenic features of most novel nidoviruses remain uncharacterized thus far (Shi et al., 2016, 2018a, b).

The groundwork for the nidovirus order was laid in the late 1980s when the first full-length genome sequences of corona-, arteri-, and toroviruses revealed striking similarities at the level of genome organization and expression. Moreover, the conservation of an array of replicase domains in these distantly related genomes pointed toward a common ancestry of the core of their replicative machinery, including the RNA-dependent RNA polymerase (RdRp) and helicase enzymes (den Boon et al., 1991). These findings were surprising at the time, in particular given the very different appearance and features of corona-, arteri-, and torovirus particles, and the large differences in genome size, which ranged from less than 13 kb for the arterivirus equine arteritis virus (EAV) to more than 31 kb for some CoVs, like MHV. The latter property placed the CoVs far apart from all other viral families characterized in the final decades of the 20th century (Gorbalenya et al., 2006). This unique position also raised questions about the processivity and fidelity of the CoV RNA-synthesizing machinery, particularly in the light of the development of the emerging RNA virus quasispecies concept and the notion that RNA virus genome sizes are constrained by the high mutation rate of their RdRp (see above).

The advent of metagenomics has taken our understanding of nidovirus host and genome diversity to the next level (Shi et al., 2016, 2018a, b; Zumla et al., 2016), even though most of these new sequences remain to be analyzed in detail and many branches of the revised nidovirus order remain sparsely populated. In terms of RNA genome size, the known upper limit increased to more than 41 kb (see above) and at the same time the former gap between the ExoN-deficient arterivirus group and the large-genome nidoviruses that contain an ExoN signature sequence has largely disappeared. Clearly, genome size unlikely is the sole factor determining the requirement for an ExoN type of proofreading function, as other factors (in particular RdRp properties) may also prominently influence replication fidelity.

**FIGURE 1:**
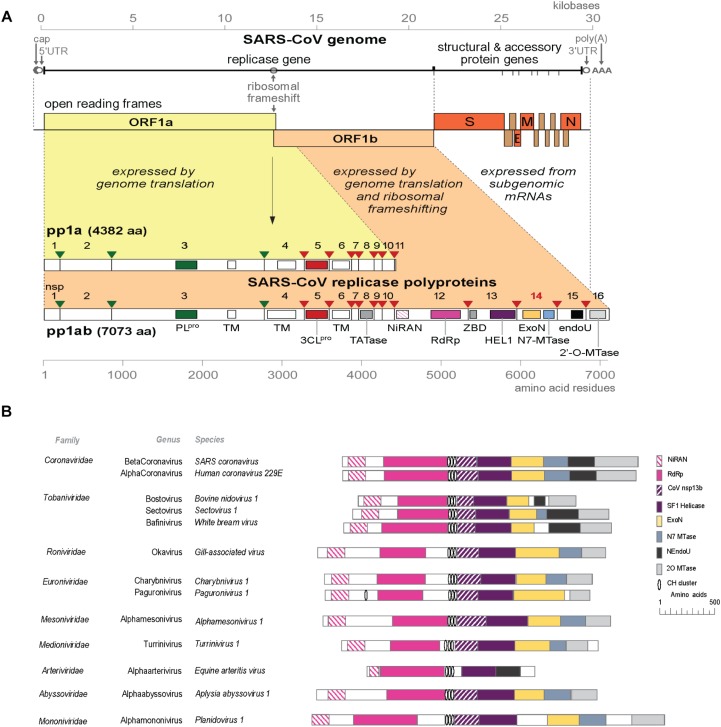
**(A)** Outline of the CoV genome organization and expression strategy. Depicted are the SARS-CoV genome and its 14 open reading frames (ORFs), i.e., the replicase ORFs 1a and 1b, the four common CoV structural protein genes (S, E, M, and N) and the ORFs encoding “accessory proteins.” The bottom half of the scheme summarizes the proteolytic processing and domain organization of the pp1a and pp1ab replicase polyproteins, the latter being produced by –1 ribosomal frameshifting. The nsp3 (PLpro, green) and nsp5 (3CLpro, red) proteases and their cognate cleavage sites are indicated in matching colors. The resulting 16 cleavage products (non-structural proteins, nsps) are indicated, as are the conserved replicase domains that are relevant for this review. See main text for references on nsp functions. Domain abbreviations and corresponding nsp numbers: PLpro, papain-like proteinase (nsp3); 3CLpro, 3C-like or main proteinase (nsp5); TM, transmembrane domain (nsp3, nsp4, and nsp6); NiRAN, nidovirus RdRp-associated nucleotidyl transferase (nsp12); RdRp, RNA-dependent RNA polymerase (nsp12); ZBD, zinc-binding domain (nsp13); HEL1, superfamily 1 helicase (nsp13); ExoN, 3′-to-5′exoribonuclease (nsp14); N7-MTase, N7-guanine methyl transferase (nsp14); endoU, uridylate-specific endoribonuclease (nsp15); 2′-*O*-MTase, 2′-*O*-methyl transferase (nsp16); UTR, untranslated region. **(B)** Comparison of the predicted domain organization in the replicase polyprotein of selected members of the nine families currently classified within the order *Nidovirales* (ICTV release 2018b). Adapted from Bukhari et al. (2018), with permission. Domains were predicted using HHPred-search (Soding, 2005; Zimmermann et al., 2018); for abbreviations see the legend to panel A. Genbank accession numbers of sequences used: SARS-coronavirus (AY274119.3); Human coronavirus 229E (AF304460.1); Bovine nidovirus 1 (KM589359.1); Sectovirus 1 (KX883637.1); White bream virus (DQ898157.1); Gill-associated virus (AF227196.1); Charybnivirus 1 (KX883628.1); Paguronivirus 1 (KX883627.1); Alphamesonivirus 1 (DQ458789.2); Turrinivirus 1 (KX883629.1); Equine arteritis virus (X53459.3); Aplysia abyssovirus (NC_040711.1); Planidovirus 1 (MH933735).

**FIGURE 2:**
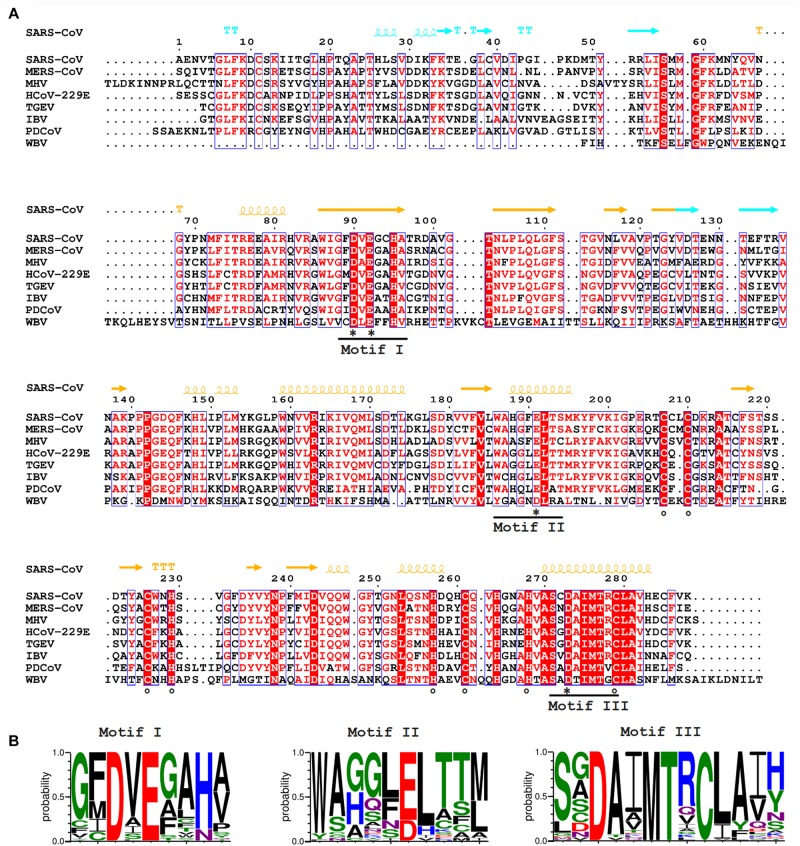
**(A)** Amino acid sequence alignment of selected nidovirus ExoN domains, including those that have been characterized experimentally and are discussed in this review, which mainly derive from members of the CoV family: SARS-CoV (NC_004718); MERS-CoV (NC_019843); HCoV-229E (NC_002645); TGEV (P0C6Y5); MHV (NP_045298); IBV (NP_040829); Porcine delta CoV (PDCoV; NC_016990), WBV (NC_008516). SARS-CoV nsp14 secondary structure (PDB: 5NFY) is indicated on top, colored according to the following domain organization: nsp10-binding site (cyan), ExoN domain (orange), hinge region (purple), N7-MTase domain (blue). Fully conserved residues are in red font and boxed, whereas partially conserved residues are displayed in red font (above 70% conservation). Catalytic residues and residues involved in formation of zinc fingers are marked with asterisks and circles, respectively. **(B)** Web-logos highlighting the three core motifs of the ExoN domain and the family of exonucleases to which it belongs.

The rapid expansion of the nidovirus order has highlighted the strict conservation of an array of five “core replicase” domains: (i) the main (or “3C-like”) protease, (ii) the nidovirus RdRp-associated nucleotidyl transferase (NiRAN), (iii) the RdRp, (iv) a Zn-binding domain (ZBD), and (v) the superfamily 1 helicase domain (HEL1), with which the ZBD is always associated (Figure 1A; Lehmann et al., 2015; Saberi et al., 2018). When present, the ExoN domain is found immediately downstream of these nidovirus-wide conserved domains, often residing in a bifunctional replicase cleavage product that also contains an N7-guanine methyltransferase (N7-MTase) activity (Chen et al., 2009; Bouvet et al., 2010), as in the case of CoV non-structural protein (nsp) 14. The size of the ExoN domain itself appears to be rather variable between different nidovirus lineages, roughly between 150 and 250 amino acid (aa) residues, depending on the presence or absence of two internal zinc finger domains [(Nga et al., 2011) and unpublished observations] (Figures 1B, 2A).

As mentioned above, for most of the novel metagenomics-derived nidoviruses we only know genome sequences, and their replication properties and enzymes have remained biologically uncharacterized thus far. This is clearly different for CoVs, which have a track record as an important group of (zoonotic) human and veterinary pathogens. With some exceptions (Durzynska et al., 2018), also the structure-function analysis of the nidoviral ExoN enzyme has been based on CoV-derived variants of the enzyme, on which we will focus our attention from this point forward.

## Coronaviral RNA*S* and Non-Structural Proteins

Like all nidoviruses, CoVs encode two very large replicase polyproteins, pp1a and pp1ab (Figure 1A), of which the latter derives from a ribosomal frameshift occurring just upstream of the ORF1a termination codon. These primary translation products of roughly 4,000–4,500 (pp1a) and 6,700–7,200 (pp1ab) residues are processed by two or three internal proteases (residing in nsp3 and nsp5) (Ziebuhr et al., 2000; Gorbalenya et al., 2006). Most of the resulting 15 or 16 nsp cleavage products assemble into a ribonucleoprotein complex that produces different types of viral RNA transcripts. In the cytoplasm of the host cell, CoV infection induces the formation of unusual double-membrane structures that are thought to support viral RNA synthesis (Gosert et al., 2002; Knoops et al., 2008). The synthesis of a nested set of subgenomic (sg) mRNAs, one of the original nidovirus hallmarks (L. *nidus* = nest), is a prominent CoV feature that drives the expression of the genes located downstream of ORF1b, encoding structural and accessory proteins (Figure 1A). An additional complexity of CoV RNA synthesis is the fact that the sg mRNAs are produced from a set of subgenome-length templates, which are both 5′ and 3′ co-terminal with the full-length negative-stranded template used for genome replication. The mechanistic details of CoV RNA synthesis and its regulation have been summarized elsewhere (Pasternak et al., 2006; Sawicki et al., 2007; Sola et al., 2015).

Over the past 25 years, CoV replicase proteins have been characterized using a combination of bioinformatics, biochemistry, structural biology, and (reverse) genetics. By now, *in vitro* biochemical assays have been described for most (predicted) replicative enzyme functions. Increasingly supported by the availability of structural information, several key CoV enzymes were probed by site-directed mutagenesis, either using *in vitro* assays or by launching engineered mutant CoV genomes from cloned cDNA templates. The CoV replicase subunits nsp7 to nsp14 and nsp16 are most intimately associated with viral RNA synthesis, either as enzymatic entity or as important co-factor [for reviews, see (Gorbalenya et al., 2006; Decroly et al., 2011b; Denison et al., 2011; Neuman et al., 2014; Sevajol et al., 2014; Snijder et al., 2016)]. Key players are the nsp12 and nsp13 subunits, which contain the RdRp and HEL domains, respectively. Each of these proteins also carries a unique N-terminal domain, NiRAN and ZBD, respectively, which both are nidovirus-specific markers whose functional importance remains to be studied in more detail (Lehmann et al., 2015; Posthuma et al., 2017; Saberi et al., 2018; Kirchdoerfer and Ward, 2019). Several nsps (nsp10, nsp14, nsp16) have been assigned functions in CoV mRNA capping and cap modification (Decroly et al., 2008, 2011a; Chen et al., 2009; Bouvet et al., 2010; Snijder et al., 2016), processes critical for both viral translation and innate immune evasion (Zust et al., 2011). Several smaller subunits, which will be discussed below, appear to act as crucial co-factors of other nsps, and such nsp-nsp interactions are also assumed to be highly relevant for the proofreading activity of the nsp14-ExoN domain (Bouvet et al., 2014; Subissi et al., 2014a; Ferron et al., 2018). Clearly, our understanding of CoV replicase activities and the assembly of the viral RNA synthesizing machinery continues to develop, which may ultimately help to explain the evolutionary success of nidoviruses at large.

## The Coronavirus RdRp in the Context of a Multimeric Enzyme Complex

The C-terminal two-thirds of the CoV nsp12 subunit are occupied by a canonical RdRp domain containing the commonly encountered motifs A to F (Gorbalenya et al., 1989; Sevajol et al., 2014; Posthuma et al., 2017; Kirchdoerfer and Ward, 2019). Conserved aspartates in motif A and in motif C presumably are responsible for the coordination of two essential metal ions in the active site (te Velthuis et al., 2010; Ahn et al., 2012; Subissi et al., 2014a). Most of our current knowledge of CoV RdRps is based on studies with SARS-CoV nsp12, which will be summarized below.

A structural prediction of the nsp12-RdRp domain was described as early as 2003 (Xu et al., 2003), but a crystal structure of the protein is still lacking. However, very recently, a cryo-EM-derived structure of SARS-CoV nps12, in a complex with two copies of nsp8 and one copy of nsp7, was reported (Kirchdoerfer and Ward, 2019). Like other + RNA viral RdRps, the CoV RdRp displays a characteristic “cupped right hand” organization, including thumb, palm and fingers subdomains (Xu et al., 2003; Sexton et al., 2016; Posthuma et al., 2017; Kirchdoerfer and Ward, 2019). A so-called “priming loop,” a typical short β-strand that is considered to be a signature for primer-dependent RNA synthesis, is lacking.

Technical challenges in obtaining stable and active recombinant nsp12 have hampered the biochemical characterization of CoV RdRp activity. Only poor enzymatic activities were observed and initially both primer-dependent RNA synthesis and *de novo* initiation were reported [reviewed in (Snijder et al., 2016; Kirchdoerfer and Ward, 2019)]. However, in the presence of the small nsp7 and nsp8 subunits, the *in vitro* primer extension activity of nsp12 could be substantially increased and *de novo* initiation was observed on a 339-nt long template corresponding the 3′-terminal sequence of the SARS-CoV genome (Subissi et al., 2014b). Recombinant SARS-CoV nsp7 and nsp8 previously had been shown to multimerize into a ring-shaped hexadecamer, which was proposed to act as a processivity factor for the nsp12-RdRp while copying the long CoV RNA genome (Zhai et al., 2005). Thus, CoV nsp12-RdRp activity was postulated to depend on the formation of a nsp7/nsp8/nsp12 tripartite complex, at least for some steps of RNA synthesis. The exact stoichiometry of this complex remains to be studied in more detail, particularly in the light of the recently published cryo-EM structure of SARS-CoV nsp12, in which the protein was complexed with a single nsp7/nsp8 dimer plus an additional nsp8 monomer rather than an nsp7-nsp8 hexadecamer (Kirchdoerfer and Ward, 2019). It should be noted that the *in vitro* RdRp activity of the latter complex remains to be demonstrated. Furthermore, for feline coronavirus (FCoV) nsp7 and nsp8, despite being structurally similar to their SARS-CoV equivalents, a higher-order complex quite different from the hexadecamer was described: a heterotrimer consisting of two copies of nsp7 and a single copy of nsp8 (Xiao et al., 2012).

Early *in vitro* assays using recombinant SARS-CoV nsp8 revealed an RNA polymerase activity typically generating products of up to six nucleotides (Imbert et al., 2006). This activity was implicated in the priming of CoV RNA synthesis, particularly in light of the (predicted) absence of a priming loop in the nsp12-RdRp domain (see above). Thus, nsp8 was proposed to act as a primase that could synthesize small oligonucleotides to be extended by the nsp12-RdRp. However, when studying the activity of the nsp7/nsp8/nsp12 tripartite complex, no *de novo* initiation by nsp8 was detected when the nsp12-RdRp domain was inactivated by a motif C D760A substitution (Subissi et al., 2014a). Most recently, an *in vitro* study employing nsp8 from human coronavirus (HCoV) 229E could not establish nsp8-associated primase or RdRp activities, but instead revealed a 3′-terminal adenylyl transferase activity that may serve to equip viral transcripts with their 3′-poly(A) tail (Tvarogova et al., 2019). Although, the importance of nsp8 as co-factor in RNA synthesis is undisputed, its interplay with nsp12 clearly remains to be investigated in more detail, in particular since the issue of nsp12 primer origin/usage seems to be wide open again.

## Timeline of Discovery and Characterization of Coronavirus ExoN

The bioinformatics-driven discovery of the nidoviral ExoN domain in 2003 was based on distant sequence similarities with cellular homologs belonging to the DEDD superfamily of exonucleases, such as the proofreading exonuclease domain of *Escherichia coli* DNA polymerase I (Snijder et al., 2003). Subsequently, the predicted 3′-to-5′ exoribonuclease activity was confirmed using *in vitro* assays with recombinant SARS-CoV nsp14 and synthetic RNA substrates (Minskaia et al., 2006). By using reverse genetics, the same authors also demonstrated that ExoN activity is critical for viability of the alphacoronavirus HCoV-229E, as inactivation of the enzyme’s active site resulted in a severe defect in overall viral RNA synthesis and a failure to recover infectious viral progeny. Shortly thereafter, strikingly different findings were obtained for the corresponding ExoN-knockout mutants of two betacoronaviruses, MHV and SARS-CoV (Eckerle et al., 2007, 2010), which are somewhat crippled, but viable in cell culture. In strong support of the original hypothesis that ExoN may act as a proofreading enzyme, ExoN inactivation was found to confer a “mutator phenotype,” as was evident from a 15- to 21-fold increase in mutation frequency – relative to the wild-type (wt) control - during replication and passaging in cell culture. The ability of ExoN to excise 3′-terminal mismatched nucleotides from a double-stranded (ds) RNA substrate was demonstrated *in vitro* using SARS-CoV nsp14 (Bouvet et al., 2010). This activity was strikingly enhanced by the addition of nsp10, suggesting the two subunits operate as a heterodimer in a mismatch repair mechanism that serves to promote the fidelity of CoV RNA synthesis. Follow-up studies from the Marseille laboratory also described the *in vitro* association of SARS-CoV nsp14 with the nsp7/nsp8/nsp12 tripartite complex (Subissi et al., 2014a) and demonstrated that ExoN can efficiently excise ribavirin 5′-monophosphate, possibly explaining why this broad-spectrum antiviral drug is poorly active against CoVs (Snijder et al., 2003; Ferron et al., 2018).

In the meantime, it had become clear that the ExoN-containing nsp14 subunit of the CoV replicase, which is about 60 kDa in size, is a bifunctional protein. A genetic screening approach in a yeast system revealed that the C-terminal domain of nsp14 exhibits (N7-guanine)-methyltransferase (N7-MTase) activity (Chen et al., 2009). Following the *in vitro* characterization of its activity, this enzyme was implicated in the N7-methylation of the (presumed) 5′-terminal cap structure of CoV mRNAs, a modification that is critical for mRNA recognition by the cellular translation machinery (Bouvet et al., 2010). The bimodular ExoN/N7-MTase organization is conserved in most nidovirus families, but the N7-MTase domain is lacking in, e.g., toroviruses, bafiniviruses (Durzynska et al., 2018) and several recently discovered nidoviruses (Figure 1B; Lauber et al., 2013; Bukhari et al., 2018; Saberi et al., 2018). The latter findings raise new questions about the mRNA capping pathway(s) employed by these particular virus groups and nidoviruses at large.

Crystal structures of SARS-CoV nsp14 in complex with its nsp10 co-factor (PDB entries 5C8U and 5NFY) revealed several unique structural and functional features (Ma et al., 2015; Ferron et al., 2018). Below we will discuss nsp14 structure and function in more detail, followed by a more extensive description of the reverse genetics data obtained with ExoN-knockout mutants of various CoVs and other functional considerations.

## Coronavirus nsp14 Harbors Exoribonuclease and N7-Methyltransferase Activities

The CoV ExoN domain was originally identified on the basis of sequence similarities with distant cellular homologs (Snijder et al., 2003) and classified into the superfamily of DEDD exonucleases, which contains the proofreading domains of many DNA polymerases as well as other eukaryotic and prokaryotic exonucleases (Zuo and Deutscher, 2001). These enzymes catalyze the excision of nucleoside monophosphates from nucleic acids in the 3′-to-5′ direction using a mechanism that depends on two divalent metal ions and a reactive water molecule (Deutscher and Marlor, 1985; Beese and Steitz, 1991; Steitz and Steitz, 1993). The name of the DEDD superfamily derives from its four conserved active site residues that are distributed over three canonical motifs (I, II, and III; Figures 2A,B) in the primary structure (Bernad et al., 1989). Originally, in SARS-CoV nsp14, residues D90/E92 (motif I), D243 (motif II), and D273 (motif III) were identified as putative active site residues (Snijder et al., 2003; Minskaia et al., 2006). Subsequently, the SARS-CoV nsp14 crystal structure revealed that ExoN in fact is a DEED enzyme as, instead of D243, E191 was identified as the acidic active site residue in motif II (Ma et al., 2015). Interestingly, when aligning ExoN sequences from different nidovirus taxa, D243 in SARS-CoV nsp14 is fully conserved, whereas the equivalent of E191 alternates between E and D (Figures 2A,B). The structural studies (Ma et al., 2015; Ferron et al., 2018) also revealed the presence of a fifth catalytic residue (H268 in motif III), identifying ExoN as a member of the DEDDh/DEEDh subfamily (Barnes et al., 1995; Zuo and Deutscher, 2001).

**FIGURE 3:**
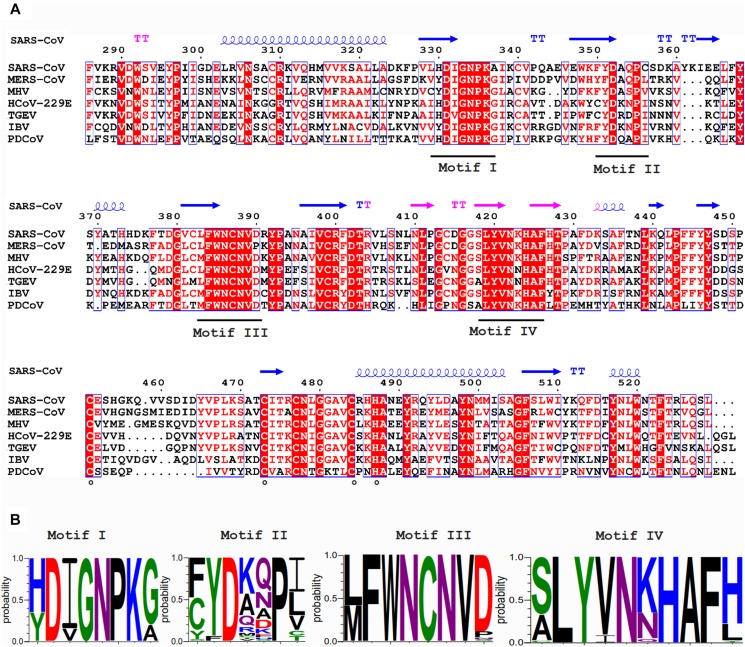
**(A)** Amino acid sequence alignment of the N7-MTase domains from the coronaviruses listed in Figure 2. See the Figure 2 legend for viruses and accession numbers used. SARS-CoV nsp14 secondary structure (PDB: 5NFY) is indicated on top, and domain colors and sequence conservation are highlighted as explained in the Figure 2 legend. Residues involved in the formation of the ZF3 zinc finger are marked with circles. **(B)** Web-logos highlighting the four most-conserved motifs of the N7-MTase domain.

**FIGURE 4:**
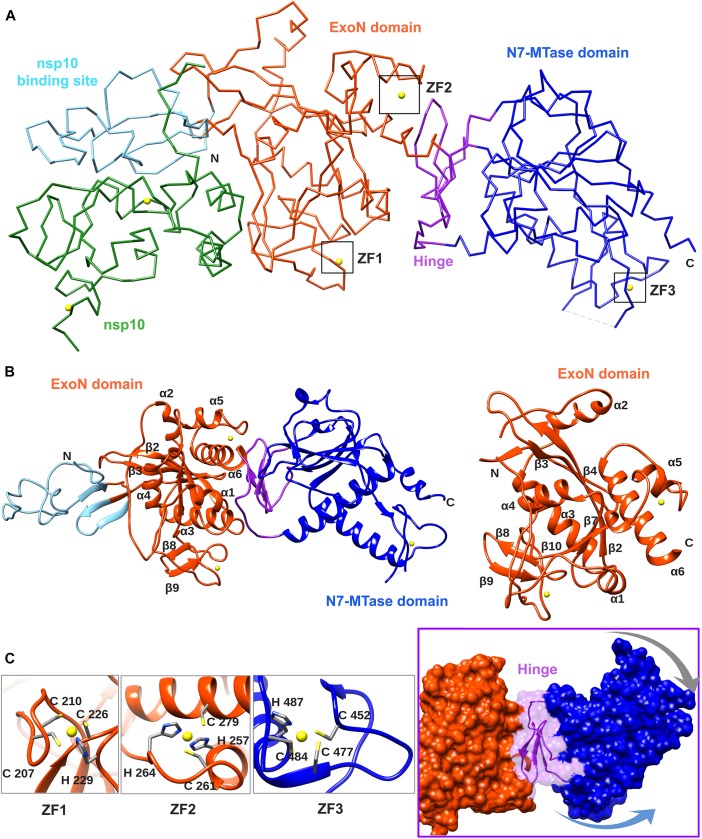
Overall structure of the SARS-CoV nsp14–nsp10 complex (PDB: 5NFY). **(A)** Cartoon representation of the crystal structure of the nsp14–nsp10 heterodimer, with domain colors used as follows: nsp10, green; nsp10-binding site of nsp14, cyan; nsp14 ExoN domain, orange; hinge region, purple; nsp14 N7-MTase domain, blue. The unresolved nsp14 residues 454–464 are represented by a dashed line. Zinc ions are shown as yellow spheres. **(B)** Cartoon representation of SARS-CoV nsp14 (left) and its ExoN domain (right), highlighting the secondary structure elements referred to in the main text. **(C)** Close-ups of the three zinc fingers (ZF) of nsp14 and the hinge region that connects the ExoN and N7-MTase domains. Arrows indicate the positional flexibility of the N7-MTase domain, which is induced by the presence of the hinge region.

In contrast to nsp14’s ExoN activity, which was inferred from bioinformatics analysis, the presence of an N7-MTase in the C-terminal domain of nsp14 was not predicted. This enzyme was discovered upon expression of TGEV and SARS-CoV nsp14 in the yeast *Saccharomyces cerevisiae*, which could rescue a mutant yeast strain lacking the native N7-MTase (Chen et al., 2009). The N7-MTase activity was further corroborated using biochemical assays with purified recombinant SARS-CoV nsp14, which was found capable of adding a methyl group to non-methylated cap analogs or GTP substrates, in the presence of *S*-adenosyl methionine (SAM) as methyl donor (Chen et al., 2009; Jin et al., 2013). Alanine scanning mutagenesis and *in vitro* assays with nsp14 highlighted two clusters of residues that are key to the MTase activity (Chen et al., 2009, 2013). The importance of the first cluster, a canonical SAM-binding motif I (DxGxPxG/A; Figures 3A,B) consisting of nsp14 residues 331–336, was confirmed by ^3^H-labeled SAM cross-linking experiments (Chen et al., 2009). The second cluster, encompassing residues 414 and 428, in the crystal structure forms a constricted pocket that holds the GTP moiety of the cap structure (GpppA) between two β-strands (β1 and β2) and helix 1 (Figure 4C). In this manner, nsp14 positions the N7 position of the cap in close proximity of the methyl donor, thus facilitating transfer by an in-line mechanism (Ma et al., 2015). Comparative sequence analysis of N7-MTase domains revealed that a number of residues crucial for substrate and ligand binding are conserved among homologous enzymes of different nidoviruses (Ma et al., 2015; Ferron et al., 2018; Saberi et al., 2018).

Biochemical analysis confirmed that the two enzymatic activities of nsp14 are functionally distinct (Chen et al., 2009) and physically independent, as also deduced from the structural studies summarized below (Ma et al., 2015; Ferron et al., 2018). However, deletions within the ExoN domain, N-terminal nsp14 truncations of between 78 and 90 amino acids, and alanine substitutions in the N-terminal domain (R84A and W86A) all drastically attenuated or completely abolished the *in vitro* N7-MTase activity (Chen et al., 2009, 2013). Although such changes may affect overall protein structure and function, these results may also indicate that the two enzymatic domains of nsp14 are structurally interconnected, with N7-MTase activity depending on the integrity of the N-terminal ExoN domain. On the other hand, the ExoN domain is not directly involved in SAM binding by the N7-MTase (Chen et al., 2009) nor does the N7-MTase activity depend on the nsp10-nsp14 interaction that strongly enhances ExoN activity (Bouvet et al., 2012).

## Structural Biology of SARS-CoV nsp14

The crystal structure of SARS-CoV nsp14 confirmed a bimodular protein composed of ExoN and N7-MTase domains that are each accompanied by an N-terminal structural domain (Figure 4A). The overall protein architecture is as follows: (i) a flexible N-terminal domain forming the nsp10 docking site, (ii) the ExoN domain, (iii) a flexible hinge region consisting of a loop and three strands, and (iv) the C-terminal N7-MTase domain (Ma et al., 2015; Ferron et al., 2018).

The CoV ExoN domain has an α/β fold reminiscent of other members of the DEDD exonuclease superfamily (Derbyshire et al., 1991). Its core is formed by a twisted central β-sheet composed of five β-strands that are flanked by five α-helices (Ma et al., 2015; Ferron et al., 2018). From this central domain, an inserted β-hairpin structure containing β5 and β6 protrudes to form with β1 a second anti-parallel β-sheet that binds to nsp10 (Figure 4B). Nsp14 interacts with nsp10 figuratively similar to a “hand (nsp14) over fist (nsp10)” conformation (Ferron et al., 2018). The fingers of the hand are formed by the flexible N-terminal region of nsp14 (residues 1–50), β1 (residues 51–55), and an antiparallel β-strand protruding from the ExoN domain (residues 122–138), while the palm is composed of residues 55–69 (top) and residues 195–202 (side) (Figure 4B). The interaction with nsp10 induces conformational changes in the N-terminal region of ExoN that modulate the distance between the catalytic residues in the back of the nsp14 palm and, consequently, impact ExoN activity (Ferron et al., 2018).

The CoV ExoN structure shares the conserved general architecture of DEDD-type exonucleases, including other proofreading ExoN domains like that of the Klenow fragment of *E. coli* DNA polymerase I, the ε subunit of DNA polymerase III (Ma et al., 2015), and another viral exonuclease (Yekwa et al., 2019), currently being peer-reviewed. On the other hand, distinguishing features are the N-terminal nsp10 interaction domain, a β-hairpin structure containing β5 and β6 that also interacts with nsp10, and two zinc finger (ZF) motifs. The first zinc finger (ZF1) is placed between α4 and β10 and formed by residues Cys207, Cys210, Cys226, and His229. The second zinc finger (ZF2), comprising residues His257, Cys261, His264, and Cys279, is located between α5 and α6 (see Figure 4C). The H268 and D273 active site residues are embedded within ZF2 (see Figure 2A), which is conserved among all nidoviruses with the exception of PSCNV (Saberi et al., 2018). Site-directed mutagenesis studies suggested that ZF1 contributes to the structural stability of nsp14, since no soluble SARS-CoV nsp14 could be obtained upon ZF1 disruption. ZF2 is important for catalysis, as replacement of important residues abolished the enzymatic activity of recombinant ExoN (Ma et al., 2015).

**FIGURE 5:**
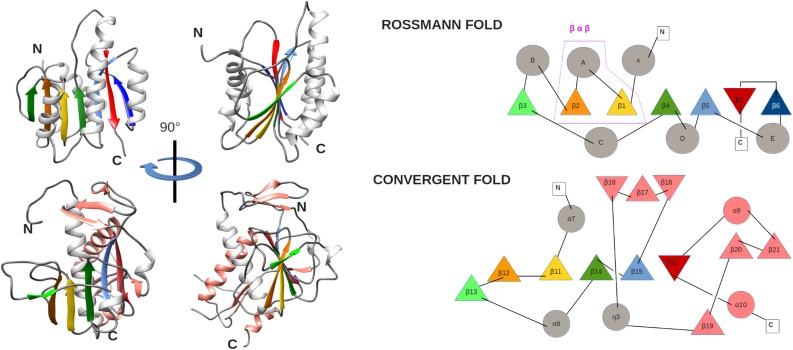
(left) Comparison of MTase ribbon models of the canonical Rossmann fold (top) of the FtsJ MTase (PDB: 1EJ0) and the convergent fold of the SARS-CoV nsp14 N7-MTase domain (bottom). Two orientations related by a 90° rotation along the vertical axis are shown. Secondary structures are colored to highlight the topology: loop (gray), α-helix (white), and β-strands (green, orange, gold, green, light blue, red), extra α-helix and β-strand of nsp14 (salmon). (right) Corresponding topology diagrams of Rossman fold MTases and the nsp14 N7-MTase domain. β-strands (triangles) and α-helices (circles) follow the same color code as for the ribbon representation in panel A. The β−α−β structural motif, which defines the Rossmann fold, is boxed.

The ExoN and N7-MTase domains of nsp14 are separated by a hinge region that is conserved across CoVs. The hinge may allow significant movement between the two enzymatic domains by allowing lateral and rotational movements of the C-terminal domain with respect to the N-terminal domain, which maybe important to coordinate nsp14’s activities (Figure 4C). The nsp14 N7-MTase domain does not exhibit the canonical Rossmann fold that is commonly found among RNA virus MTases or other RNA cap 0 MTases (Byszewska et al., 2014; Chouhan et al., 2019) and does not belong to any of the five classes of SAM-dependent MTases (Schubert et al., 2003), adding another dimension to the unique structural features of this bifunctional protein (Figure 5; Ferron et al., 2018). In general, the Rossmann fold follows a characteristic β-α-β architecture with seven parallel hydrogen-bonded β-strands composing the core of the β-sheet structure, with at least three α-helices on each side (Rao and Rossmann, 1973; Ferron et al., 2018). The nsp14 N7-MTase comprises a total of 12 β-strands and five α-helices, with the central β-sheet being composed of five β-helices instead of seven. Additionally, the N7-MTase domain ends with an α-helix, α10, a modification that stabilizes the local hydrophobic environment and is found in SAM-dependent MTases (Martin and McMillan, 2002). A ZF motif (ZF3) consisting of C452, C477, C484, and H487 is located between strand β21 and helix α9 and is important for the proper folding of this region (Figures 4C, 5). The three ZF motifs are a specific structural signature of nsp14.

## Biochemical Support for ExoN-Driven Error Correction

The first biochemical assays with purified SARS-CoV nsp14 demonstrated the capability to hydrolyze both double-stranded (ds) and single-stranded (ss) RNA substrates in the 3′-to-5′ direction, with a preference for dsRNA substrates (Minskaia et al., 2006). ExoN activity was not found to be RNA sequence-specific, but DNA substrates were not degraded. Ribonuclease activity depended on the presence of divalent metal ions, and was strongly reduced or lost upon substitution of the predicted catalytic residues in motifs I, II, or III with alanine (Minskaia et al., 2006). Whereas basal ExoN activity does not require the presence of co-factors (Minskaia et al., 2006; Chen et al., 2007), Bouvet et al. (2012) demonstrated that SARS-CoV ExoN activity is enhanced > 35-fold in the presence of nsp10, a small ORF1a-encoded subunit of the CoV replicase that also serves as a co-factor for nsp16’s 2′-*O*-methyltransferase (2′O-MTase) activity (Bouvet et al., 2010). Mutagenesis of nsp10 surface residues into alanine in many cases disrupted the interaction with nsp14, leading to a significant reduction of ExoN activity and the conclusion that nsp14 and nsp16 share a common interaction surface on nsp10 (Bouvet et al., 2012, 2014). The nsp10/nsp14 complex preferentially degrades dsRNA substrates suggesting that heterodimerization does not change ExoN’s substrate specificity (Bouvet et al., 2012). Furthermore, RNA substrates with a 3′-end duplex structure (like a stable hairpin) and fully base-paired RNA substrates were also efficiently hydrolyzed by the nsp10/nsp14 complex (Bouvet et al., 2012). Importantly, in an assay intended to mimic RdRp incorporation errors, the SARS-CoV nsp10/nsp14 complex was able to excise 3′-terminal mismatched nucleotides from a dsRNA substrate (Bouvet et al., 2012). A more elaborate analysis of ExoN substrate use and specificity suggested that catalysis is determined by the presence of mismatches rather than the nature of the misincorporated nucleotide (Bouvet et al., 2012; Ferron et al., 2018). However, when the stretch of 3′-terminal mismatched nucleotides was increased beyond 2 nucleotides, a sharp decrease of excision activity was observed (Bouvet et al., 2012). Comparable *in vitro* mismatch excision activity was reported recently for the ExoN domain of the bafinivirus white bream virus (WBV), using dsRNA substrates containing up to three mismatches (Durzynska et al., 2018). These assays were performed using WBV’s nsp14 equivalent alone, as no nsp10 homolog seems to be encoded by bafiniviruses. Thus far, this study constitutes the only description of *in vitro* ExoN activity for a nidovirus that does not belong to the CoV family.

The above studies provided the first biochemical evidence that ExoN, likely in concert with the tripartite RdRp complex (nsp7/nsp8/nsp12 in CoVs), may function as a proofreading enzyme that preferentially targets 3′-terminal single mismatches. Indeed, SARS-CoV nsp14 and the nsp7/nsp8/nsp12 complex were able to associate with each other, while retaining their RNA synthesis, N7-MTase, and exoribonuclease activities (Subissi et al., 2014b). Although, the structural basis of the interaction(s) between nsp14 and the nsp12-RdRp domain remains to be elucidated, *in vitro* studies revealed that both the ExoN and N7-MTase domains of nsp14 are involved (Ferron et al., 2018). Recent biochemical studies provided more insight into the interplay between the SARS-CoV nsp7/nsp8/nsp12 RdRp complex and the nsp10/14 heterodimer (Ferron et al., 2018). Using a primer-template substrate containing an A:A mismatch at the 3′ end of the primer, extension of the primer was barely observed, suggesting that the SARS-CoV RdRp encounters a kinetic block to extend a substrate with a 3′-terminal mismatch (Ferron et al., 2018). Strikingly, addition of nsp10/nsp14 to this assay appeared to relieve these constraints and full-length polymerization products were observed, suggesting that ExoN had removed the A:A mismatch before polymerization was resumed. Sequencing of RNA products revealed that 90% had the corrected sequence (Ferron et al., 2018). Similarly, ribavirin 5′-monophosphate (a guanosine analog) was efficiently excised from RNA substrates in the presence of nsp14 or nsp10/nsp14 (Ferron et al., 2018).

Together with the mutator phenotype observed for ExoN-knockout mutants of SARS-CoV and MHV (see below), the above data strongly suggest that ExoN contributes to the fidelity of CoV RNA synthesis. It is striking, that *in vitro* single-nucleotide incorporation assays the SARS-CoV RdRp complex (nsp7/nsp8/nsp12, without nsp14) displayed a lower fidelity than the RdRp of dengue virus, a flavivirus with a three-fold smaller genome (Ferron et al., 2018). Clearly, a direct *in vitro* comparison of the properties of (distantly related) viral RdRps is not straightforward. Moreover, our perception is “fragmented” (at best…) when it comes to the biochemical evolution of RdRp and ExoN features following the postulated acquisition of the latter by an ancestral nidovirus (Snijder et al., 2003; Nga et al., 2011). ExoN acquisition may indeed have facilitated genome expansion, but - to an unknown extent - it may also have relaxed the nucleotide selectivity of the RdRp, and therefore the fidelity of RNA synthesis, which would be in line with the biochemical observations outlined above (Ferron et al., 2018). This would also leave space for the possibility that nidoviruses may exist which combine the use of ExoN with an intrinsic RdRp fidelity that is substantially higher than that observed for present-day CoVs. In fact, also this scenario may have contributed to expand nidoviral genomes to the currently known upper limit (41.1 kb for PSCNV) (Saberi et al., 2018) and (potentially) beyond. In this light, it would be highly interesting to perform similar *in vitro* assays to establish and compare the intrinsic RdRp fidelity of diverse nidoviruses, including those with the longest genomes and those naturally lacking an ExoN domain (Ferron et al., 2018). Subsequently, nucleotide incorporation assays combining RdRps and their cognate ExoN may provide important insights into the biochemical synergy of the two enzymes, and may ultimately allow us to correlate intrinsic RdRp fidelity and ExoN activity across the broad spectrum of viruses included in the nidovirus order.

## Characterization of Coronavirus ExoN-Knockout Mutants

The study from the Ziebuhr laboratory that demonstrated SARS-CoV nsp14 *in vitro* exoribonuclease activity (Minskaia et al., 2006) also described the first engineered ExoN-knockout CoV mutants. However, for biosafety reasons, these experiments were performed using the alphacoronavirus HCoV-229E rather than the betacoronavirus SARS-CoV. The intracellular accumulation of HCoV-229E RNA was found to be severely reduced (by approximately 2 log) for several ExoN active site mutants in motifs I, II, and III. Moreover, relative molar ratios of sg mRNAs were altered and the production of alternative sg transcripts was suspected, based on an analysis of the low amounts of viral RNA produced in transfected cells. Most importantly, infectious virus progeny could not be recovered from the medium of cells transfected with full-length RNA carrying ExoN-inactivating mutations, and consequently ExoN activity was concluded to be critical for HCoV-229E replication (Minskaia et al., 2006).

If SARS-CoV rather than HCoV-229E had been the subject of this initial reverse genetics study, the conclusions would have been quite different. Subsequent work from the Denison and Baric laboratories showed that the replication of equivalent ExoN-knockout mutants of SARS-CoV and MHV was affected but certainly not abolished (Eckerle et al., 2007, 2010). This was the case when substituting conserved acidic residues of either motif I (D90/E92 in SARS-CoV; mutant ExoN1) or motif III (D273, mutant ExoN3) by alanine. For MHV, these mutations reduced overall viral RNA synthesis (by 75–90% for both genomic and sg RNA) and delayed replication (by several hours), whereas also a specific change in sg mRNA synthesis was observed (reduced mRNA2 production). In MHV, progeny titers of ExoN-knockout mutants were reduced up to 1 log, with plaque sizes of ExoN-knockout mutants also showing extreme heterogeneity (Eckerle et al., 2007). For the corresponding SARS-CoV mutants, progeny titers were about fourfold reduced without a clear overall change in replication kinetics (Eckerle et al., 2007), although intracellular RNA synthesis was not studied in detail. Upon serial passaging in cultured cells, sequence analysis using both conventional and next-generation techniques revealed that the genomes of ExoN-knockout MHV and SARS-CoV mutants accumulated up to 21-fold more mutations than their parental controls, thus providing direct experimental evidence for a connection between ExoN activity and CoV replication fidelity (Eckerle et al., 2007, 2010).

To investigate this “mutator phenotype” *in vivo*, mutant ExoN1 was engineered in a mouse-adapted (MA) SARS-CoV backbone (Graham et al., 2012). During its *in vitro* characterization, this mutant’s progeny titers were reduced by less than 1 log. Quantitative RT-PCR data indicated that the accumulation of wild-type and mutant genome initially was roughly equivalent (6 h p.i.), but that the mutant genome accumulated to about 10-fold lower levels later in infection (24 h p.i.), possibly due to the rapid accumulation of unfavorable mutations (Graham et al., 2012). Similar to results obtained upon passaging in cell culture, the ExoN1-MA SARS-CoV mutant exhibited an 11.5-fold increased mutation frequency. The virulence of this mutant was attenuated, resulting in (strongly) reduced disease and expedited virus clearance (Graham et al., 2012). Long-term persistent infection of SCID mice allowed a comparison of mutational loads after 30 days, revealing 9.6-fold more mutations across the genome for the progeny of ExoN-deficient MA SARS-CoV.

As expected, due to their reduced replication fidelity and/or impaired overall replication capacity, MHV and SARS-CoV ExoN mutants display increased sensitivity to mutagenic agents like the nucleoside analog 5-fluorouracil (5-FU) (Smith et al., 2013). Similarly, an MHV mutant in which the interaction of nsp14 with its nsp10 co-factor was predicted to be disturbed by two mutations (R80A/E82A in nsp10) was more sensitive to 5-FU than wt virus (Smith et al., 2015). Although this finding could be taken as further support for the hypothesis that it is the nsp10/nsp14 complex that acts as a proofreading enzyme, the situation likely is more complex. Specifically, a single alanine substitution at one of the nsp10 positions targeted for MHV proved to be lethal in SARS-CoV (mutant nsp10-H80A) (Bouvet et al., 2014). Also other nsp10 mutations that disturb the interaction with nsp14 were lethal, and thus had a much stronger impact on SARS-CoV viability than the direct inactivation of ExoN’s enzymatic activity (Bouvet et al., 2014). Taking into account that the nsp10/nsp14 and nsp10/nsp16 interaction regions overlap (Ferron et al., 2018), it can be hypothesized that some of the nsp10 mutations interfere with the activities of both complexes. Clearly, this raises important questions about the (multi)functionality of nsp10 and/or the nsp10-nsp14 complex in CoV replication.

Interestingly, and in spite of its reduced replication fidelity, reversion of the MHV-ExoN1 mutant was not reported thus far, even when it was serially passaged in cell culture 250 times (Graepel et al., 2017). However, over this long period of time, the passaged mutant virus exhibited an eightfold higher mutation frequency and accumulated a variety of adaptive non-synonymous mutations. These were spread across the genome and partially compensated for the replication defect and decreased mutagen sensitivity, possibly by improving RdRp fidelity or increasing “mutational robustness” (Graepel et al., 2017). These compensatory mutations mapped to the nsp12-RdRp domain and to nsp14 itself, but also to subunits like nsp8, nsp9, and the nsp13 helicase domain. Full reversion of the ExoN1 mutations (DE→AA) would require a total of four nucleotide substitutions, but neither full nor partial reversion was observed, suggesting a narrow evolutionary pathway to reversion. It was proposed that replacement of only one of the active site residues suffices to minimize ExoN activity, as observed for the 3′-to-5′ exonuclease of *E. coli* polymerase I (Derbyshire et al., 1991), and that reversion at just one of the two motif I sites offers no selective advantage compared to the double mutant (Graepel et al., 2017).

In view of the replication competence of MHV and SARS-CoV ExoN-knockout mutants summarized above, it is striking that equivalent mutants proved to be non-viable in at least three other CoVs: HCoV-229E (Minskaia et al., 2006), transmissible gastroenteritis virus (TGEV) (Becares et al., 2016), and – most recently – also MERS-CoV, according to an extensive mutagenesis study from our own laboratory. Using a replicon system for the alphacoronavirus TGEV, genome replication and sg mRNA synthesis were found to be only modestly reduced upon mutagenesis of ExoN active site residues (Becares et al., 2016). Interestingly, a Cys-to-His change of the second Zn-coordinating residue of ZF1 (residue C210), severely affected sg mRNA synthesis while only mildly affecting genome replication. Upon introduction into the full-length TGEV genome, mutations equivalent to those in the SARS-CoV ExoN1 and ExoN3 mutants prevented the recovery of infectious progeny, with quantitative RT-PCR assays indicating a ∼15-fold reduction in the accumulation of genomic RNA. Additionally, a second ZF1 mutation, His-to-Cys at the position of the fourth Zn-coordinating residue (H229), did not strongly affect TGEV RNA synthesis or progeny titers, but was reported to trigger a weaker TGEV-induced antiviral response. This was attributed to a reduced accumulation of viral dsRNA, an important pathogen-associated molecular pattern (PAMP) that is recognized by innate immune sensors, which triggered a decrease of IFN-β mRNA synthesis and of IFN-induced immune factors in cell culture (Becares et al., 2016). Unfortunately, no information is available on the enzymatic activity or replication fidelity of the two TGEV ExoN ZF1 mutants, which would be required to better understand the interesting phenotype of these mutants and their capability to modulate innate immune responses. The assumption that the viable ZF1 mutant (H229C) possesses increased ExoN activity, which could explain the reduced levels of dsRNA in infected cells, seems premature. Alternative explanations for this phenotype include changes in the efficiency or kinetics of RNA synthesis. Moreover, the reported reduction of viral double-stranded RNA accumulation by this mutant should be interpreted with caution, as this conclusion was based solely on the *in situ* immunodetection of dsRNA using a monoclonal antibody with a poorly defined specificity for CoV dsRNA replication intermediates. For example, it remains unknown how changes in the protein composition or subcellular localization of the RNA-synthesizing complex may affect the accessibility of dsRNA epitopes during such immunolabeling experiments.

The results recently obtained with ExoN-knockout mutants MERS-CoV are equally intriguing, particularly since MERS-CoV is a betacoronavirus, like MHV and SARS-CoV. Using an elaborate set of ExoN active site mutants, carrying conservative or alanine substitutions, it was found that ExoN inactivation is lethal in MERS-CoV and that no sign of viral RNA synthesis can be discovered in cells transfected with these mutants full-length RNA. Again, these observations suggest that the ExoN domain and/or nsp14 (also) play a more direct and basic role in CoV RNA synthesis than merely safeguarding the long-term fidelity of replication.

## The Remarkable Phenotypic Variation Among ExoN-Knockout Mutants

As summarized above, depending on the CoV studied, the impact of ExoN inactivation on viral RNA synthesis ranges from a complete block (MERS-CoV) to various degrees of impairment, with residual RNA production supporting the generation of infectious progeny only in the case of MHV and SARS-CoV. For both these betacoronaviruses, in spite of their “mutator phenotype,” the long-term consequences of ExoN inactivation seem limited during propagation in cell culture. Viral RNA synthesis might indeed be expected to tolerate, at least to a certain extent, the inactivation of a proofreading activity that was postulated to not be directly required for RdRp activity, but to merely boost the overall quality of the replication process. Clearly, when replicating in the absence of a functional ExoN, deleterious mutations would first have to accumulate before viral fitness would begin to decrease. This does not appear to be the case for a third betacoronavirus, MERS-CoV, and for two alphacoronaviruses, HCoV-229E and TGEV, for which the immediate (full to strong) disruption of viral RNA synthesis was observed when ExoN-knockout mutants were launched by transfection of full-length RNA or DNA. In our opinion, technical variations are unlikely to explain these viability differences: with the exception of TGEV, ExoN knockout mutants were commonly launched by electroporation of *in vitro* produced full-length RNA into the cytosol of BHK-21 cells, thus providing an equal environment for the first, critical rounds of replication in a cell line that is known to be compromised in its innate immune response (Lam et al., 2005; Habjan et al., 2008). During our studies with the non-viable MERS-CoV ExoN mutants, we attempted to amplify progeny virus released from transfected BHK-21 cells in both immune-competent and -incompetent cells (e.g., Huh7 and Vero cells, respectively) with an equally negative outcome. Thus, in addition to proofreading, ExoN somehow appears to play a more basic role in the functionality of the CoV RNA-synthesizing machinery, by virtue of its exoribonuclease activity, as a domain of the bifunctional nsp14 subunit, and/or as an interaction partner for other nsps in the viral RNA-synthesizing machinery.

Among the CoVs investigated thus far, MHV and SARS-CoV (in our experience) do stand out as the two viruses exhibiting the most robust RNA synthesis and overall replication in cultured cells. Possibly, the replication activity of ExoN-deficient mutants somehow needs to cross a certain “threshold” to result in infectious progeny, and for ExoN-deficient mutants this is only achieved with the CoVs that replicate most efficiently. However, when considering the phenotypic differences of knockout mutants, in terms of virus viability and sensitivity to mutagenic agents in cell culture, it remains difficult to reconcile the reported 1- to 2-log reduction of progeny titers for ExoN-knockout MHV and SARS-CoV with the complete loss of infectious progeny reported for the ExoN-knockout mutants of various other CoVs. It is also relevant to consider the fact that low levels of residual enzymatic activity of ExoN active site mutants may go unnoticed in biochemical assays, but could still support a certain level of replication when launching the RNA of an ExoN-knockout CoV mutant. Despite the conservation of ExoN among CoVs and most other nidoviruses, the extent to which particular mutations affect enzymatic activity can only be assessed when studying these specific viral proteins in a biochemical assay (Becares et al., 2016; Case et al., 2016, 2018).

Interactions with the host’s innate immune system have been suggested to co-determine the phenotype of ExoN-knockout CoV mutants (Kindler and Thiel, 2014; Becares et al., 2016; Case et al., 2018). It has been proposed that CoV nsp14, by virtue of its ExoN activity, may counteract innate responses by degrading dsRNA replication intermediates in a similar manner as documented for the ExoN domain of the arenavirus nucleoprotein (Hastie et al., 2011; Russier et al., 2014). As CoVs employ a range of innate immune evasion mechanisms (Kindler and Thiel, 2014), it is difficult to study the importance of any single mechanism in a straightforward manner, as other innate immune antagonists will continue to operate in cells infected with mutants lacking one particular immune evasion function.

Case et al. (2018) showed that MHV ExoN(−) virus is sensitive to IFN-β, and that its replication is strongly attenuated in innate immune-competent bone marrow-derived macrophages (BMMs), an effect that was partially restored in interferon-alpha/beta receptor knockout (IFNAR−/−) BMMs. However, upon infection with the MHV-ExoN 1 mutant, neither upregulation of interferon mRNA expression nor induction of the OAS/RNAseL or PKR pathway was observed, in contrast to what would be expected if nsp14 would indeed degrade a PAMP like viral dsRNA. The MHV ExoN1 mutant yielded progeny with a ∼10-fold reduced specific infectivity and decreased relative fitness. This property was attributed to the lack of ExoN activity, but the mechanisms underlying the reduced fitness and altered IFN sensitivity remain to be investigated (Case et al., 2018). As summarized in the previous paragraph, TGEV ExoN active site mutants were non-viable (see also above), but an nsp14 mutant carrying a ZF1 mutation (H229C; close to the interaction region with nsp10) was reported to accumulate less dsRNA in infected cells and trigger a weaker antiviral response (Becares et al., 2016). These results could be taken to suggest that ExoN may modulate innate immune responses, but TGEV nsp14 remains to be characterized biochemically and for now one can only speculate about the level of ExoN activity of this particular mutant.

Several cellular interferon-stimulated gene products have been implicated in the hypermutation of viral genomes, so it remains to be established how directly the properties of ExoN mutants are determined by a lack (Case et al., 2018) or surmised increase of exoribonuclease activity (Becares et al., 2016). An additional functional consideration is the fact that the bulk of CoV dsRNA replication intermediates were found to be confined to peculiar double-membrane vesicles, which are part of the CoV replication organelle that drives viral RNA synthesis in infected cells (Knoops et al., 2008; Neuman, 2013). This feature, which in itself has been proposed to be an innate immune evasion strategy, would potentially complicate access of nsp14 to viral dsRNA substrates.

## Nsp14: An Attractive Target for Antiviral Drug Design?

Currently, there are no FDA-approved antiviral drugs for the treatment of CoVs, which is mainly due to limited interest from the side of the pharmaceutical industry, despite the loss of human lives during the short-lived SARS outbreak and the continuing MERS epidemic. Moreover, antiviral hits identified so far often suffered from poor selectivity indexes. Drug development efforts were further restricted by the limitations of available animal models and potency failure in clinical trials (Zumla et al., 2016). Taking into account the combination of ExoN and N7-MTase activities in a single protein, and its importance in viral replication, CoV nsp14 is an attractive target for antiviral drugs. Thus far, only two classes of compounds that (in)directly interfere with its activities have been analyzed in more detail: nucleoside analogs and methyltransferase inhibitors.

Nucleoside analogs can have different mechanisms of action. They may interfere with RNA synthesis directly (for instance by obligate chain termination) or may inhibit virus replication indirectly, for example by inducing lethal mutagenesis or perturbing intracellular nucleoside triphosphate pools. The ExoN proofreading function might counteract these compounds mode of action and, in order to circumvent this, a nucleoside would need to be incorporated more efficiently than it will be excised by ExoN, or should be resistant to ExoN-mediated removal (Crotty et al., 2000; Smith et al., 2013; Warren et al., 2016; Agostini et al., 2018). Recently, in spite of these potential complications, GS-5734 (Remdesivir, a monophosphoramidate prodrug of an adenosine analog) was shown to be a potent inhibitor of the replication of human and zoonotic CoVs *in vivo* and *in vitro* (Warren et al., 2016; Sheahan et al., 2017; Agostini et al., 2018). Compared to the wt control, replication of the MHV ExoN1-knockout mutant was inhibited more efficiently by GS-5734, suggesting that the compound’s activity is limited by ExoN’s capability to excise and remove it after its incorporation into the RNA chain by the viral RdRp. The simultaneous targeting of RdRp and ExoN functionality with a combination of a nucleoside analog and a specific exoribonuclease inhibitor may also be worth exploring. In the case of nucleoside analogs like ribavirin, such an approach may even restore antiviral efficacy against CoVs and other viruses equipped with a proofreading mechanism (Ferron et al., 2018).

Regarding the inhibition of the nsp14 N7-MTase, only a few compounds have been identified that inhibit its activity *in vitro*: adenosylhomocysteine, aurintricarboxylic acid (ATA), and sinefugin (Bouvet et al., 2010; Sun et al., 2014; Aouadi et al., 2017). Further work is needed to optimize these hits in order to study their activity *in vivo*, and investigate their specificity for this viral enzyme. Taking into account the unique fold of the N7-MTase enzyme compared to other MTases, this might facilitate the drug development of specific compounds targeting this domain (Ma et al., 2015; Ferron et al., 2018).

## Conclusion and Future Directions

The order *Nidovirales* constitutes a + RNA virus lineage displaying a unique combination of molecular biological features, including a genome size that ranges from “somewhat above average” (arteriviruses, 12–16 kb) to the largest RNA virus genomes currently known (PSCNV, 41 kb). Upon its discovery, promoted by the relationship with other proofreading exonucleases, the ExoN domain was postulated to have played an important role in nidoviral evolution and genome expansion (Snijder et al., 2003) by providing a proofreading activity that enhances the replication fidelity. Indeed, now that the nidovirus order has grown substantially over the past decade, the conservation of ExoN across a wide range of distantly related nidoviruses with genome sizes above 18 kb testifies to the important role this enzyme must play. Consequently, this role was incorporated in an advanced theoretical model of nidoviral genome dynamics (Lauber et al., 2013; Saberi et al., 2018), in which the ancestral expansion of ORF1b, which includes the ExoN domain, facilitated the subsequent growth of other parts of the genome. In parallel, experimental evidence has accumulated, mainly derived from studies with the well-studied betacoronaviruses SARS-CoV and MHV, leaving little doubt about the involvement of ExoN in fidelity control during genome replication (Minskaia et al., 2006; Eckerle et al., 2007, 2010; Bouvet et al., 2012; Graham et al., 2012; Ferron et al., 2018).

While the increasing genome size upper limit and the discovery of a proofreading mechanism constitute clear and exciting paradigm shifts in RNA virology, important questions regarding ExoN function and importance remain to be resolved. Among these, the wide phenotypic variation among the ExoN-knockout mutants of the different CoV species studied thus far (see above) is a remarkable issue. In this case, it appears to be particularly challenging to integrate the results from biochemistry, structural biology, (reverse) genetics, and the analysis of CoV-infected cells into a coherent model of ExoN function. It might also be instructive to reassess the increased mutation frequency and evolution of ExoN-knockout mutants using more advanced deep-sequencing methods that have been developed during recent years (Acevedo et al., 2014).

Although most of the ORF1b-encoded key replicative enzymes of CoVs (nsp12, nsp13, and nsp14) and their co-factors (nsp7, nsp8, and nsp10) now have been characterized *in vitro*, it is still quite unclear how these findings can be extrapolated to the viral enzyme complex in the infected cell (Ulferts et al., 2011; Snijder et al., 2016). The impressive long-term passaging experiment with the MHV ExoN1 mutant (Graepel et al., 2017) nicely illustrates the complexity and plasticity of the CoV replication machinery, documenting how a network of compensatory mutations in a variety of other nsps can – in the long run – help the virus to survive and circumvent an ExoN activity defect. Unfortunately, such studies are technically challenging or impossible for CoVs yielding non-viable ExoN-knockout mutants. In this context, it is necessary to expand the biochemical and structural characterization of CoV replicative enzymes, including ExoN, to other CoV species than SARS-CoV.

Further elucidation of the structure-function interplay between ExoN and other (viral and/or host) members of the CoV replication machinery will be key to understanding their role in viral RNA synthesis, immune evasion and pathogenesis. Such information will contribute to the design of new antiviral approaches, or the improvement of existing ones, including those relying on inducing “lethal mutagenesis” (Ferron et al., 2018). Likewise, it will allow a better assessment of the applicability of ExoN inactivation as a broad strategy for designing live-attenuated vaccines against CoVs or other nidoviruses (Graham et al., 2012), which – in terms of vaccine production – clearly depends on the viability of ExoN-knockout mutants. In this context, it would be highly interesting to explore the partial inactivation of ExoN in CoVs for which full inactivation was proven to be lethal. Now that metagenomics studies have informed us about the evolutionary success and remarkably broad host range of nidoviruses, it is important, more than ever before, to enhance our preparedness and design strategies to counter nidoviruses that are likely to emerge in human or animal host populations.

## Author Contributions

NO, CP, and ES conceived, wrote, and edited this review. FF, ED, and BC revised and extended the parts of the text – specifically in the areas of the biochemistry and structural biology, and provided various other useful suggestions and contributions. FF prepared Figures 2–5. ES and NO prepared Figure 1A. NO and ES prepared the final version of the manuscript, which was approved by all authors.

## Conflict of Interest Statement

The authors declare that the research was conducted in the absence of any commercial or financial relationships that could be construed as a potential conflict of interest.

## Footnotes

1 ICTV website: https://talk.ictvonline.org/taxonomy/
